# Caffeine inhibits hypoxia-induced renal fibroblast activation by antioxidant mechanism

**DOI:** 10.1080/19336918.2019.1638691

**Published:** 2019-07-16

**Authors:** Angkhana Nilnumkhum, Rattiyaporn Kanlaya, Sunisa Yoodee, Visith Thongboonkerd

**Affiliations:** Medical Proteomics Unit, Office for Research and Development, Faculty of Medicine Siriraj Hospital, Mahidol University, Bangkok, Thailand

**Keywords:** Catalase, coffee, fibrogenesis, hypoxia, Nrf2, renal fibrosis, ROS

## Abstract

Caffeine has been demonstrated to possess anti-fibrotic activity against liver fibrosis. However, its role in renal fibrosis remained unclear. This study investigated the effects of caffeine on renal fibroblast activation induced by hypoxia (one of the inducers for renal fibrosis). BHK-21 fibroblasts were cultured under normoxia or hypoxia with or without caffeine treatment. Hypoxia increased levels of fibronectin, α-smooth muscle actin, actin stress fibers, intracellular reactive oxygen species (ROS), and oxidized proteins. However, caffeine successfully preserved all these activated fibroblast markers to their basal levels. Cellular catalase activity was dropped under hypoxic condition but could be reactivated by caffeine. *Hif1a* gene and stress-responsive Nrf2 signaling molecule were elevated/activated by hypoxia, but only Nrf2 could be partially recovered by caffeine. These data suggest that caffeine exhibits anti-fibrotic effect against hypoxia-induced renal fibroblast activation through its antioxidant property to eliminate intracellular ROS, at least in part, via downstream catalase and Nrf2 mechanisms.

## Introduction

Chronic kidney disease (CKD) is still a major health concern worldwide. It is well known that hypertension and diabetes mellitus are the major etiologies of CKD, which is histopathologically characterized by interstitial inflammation, tubular atrophy, and fibrosis [1]. Renal fibrosis has been considered as an aberration of wound/tissue healing process, in which there is a progression rather than improvement of scar formation after renal tissue injury, and fibroblasts play a crucial role in this phenomenon [2]. Fibroblasts are quiescent cells residing in the renal interstitium that sustain renal tissue structure [3]. They are the major cells responsible for synthesis and turnover of extracellular matrix (ECM) during normal development and adaptive homeostasis after cell/tissue injury [3]. Aberrant regulation of ECM homeostasis is, therefore, an underlying mechanism for the development of renal fibrosis. As a consequence, fibroblasts are differentiated into their active phenotype (known as myofibroblasts), which have been defined by an increase of α-smooth muscle actin (α-SMA) and accumulation of the excessive amount of ECM constituents [4]. Additionally, actin filaments turn into stress fiber bundles during the fibroblast activation [2].

Hypoxia is associated with low oxygen tension involving an inadequate level of oxygen supply for maintaining cellular function and is one of the inducers for renal fibrosis as evidenced by the induction of *de novo* synthesis of α-SMA and augmentation of ECM deposition [5,6]. During progressive fibrosis, hypoxia contributes to persistent injury, leading to recruitment of inflammatory and cytokine factors, which subsequently activate fibroblasts [7]. Prolonged hypoxia generates a vicious cycle of such effects that are of great importance in contribution to renal fibrosis, which in turn aggravates renal hypoxia by limiting oxygen diffusion and thereby progression of CKD [8]. Hypoxia can also induce expression of α-SMA to activate fibroblasts to myofibroblasts that contribute directly to fibrogenesis through the increment of ECM synthesis [9,10].

Caffeine (1,3,7-trimethylpurine-2,6-dione) is a purine, methylxanthine alkaloid that mainly presents in the coffee bean. It is a pharmacologically active compound that affects the central nervous, cardiovascular, and respiratory systems with evidence of lower risk of mortality caused by heart disease, infection, stroke, and respiratory disorder in coffee consumers [11,12]. In addition, several studies have highlighted the beneficial effects of caffeine in habitual coffee drinkers to prevent liver diseases, including hepatic cancers, alcoholic cirrhosis, and hepatitis C-induced liver fibrosis [13–15]. A previous study has shown the anti-fibrotic effect of caffeine against diethylnitrosamine (DEN)- and carbon tetrachloride (CCl_4_)-induced liver fibrosis [13]. In a model of alcohol-induced liver fibrosis, activation of hepatic stellate cells via cAMP/PKA/CREB pathway was inhibited by caffeine treatment [14]. In addition, caffeine could inhibit TGF-β-induced epithelial cell and fibroblast activation *in vitro* and could also reduce collagen production in established fibrosis of lung tissue *ex vivo* [16]. However, the role of caffeine in renal fibrosis remained unclear. This study, therefore, investigated the potential effects of caffeine in renal fibrosis using hypoxia-induced fibroblast activation as a study model.

## Results

### Validation of the study model

**Figure 1.**
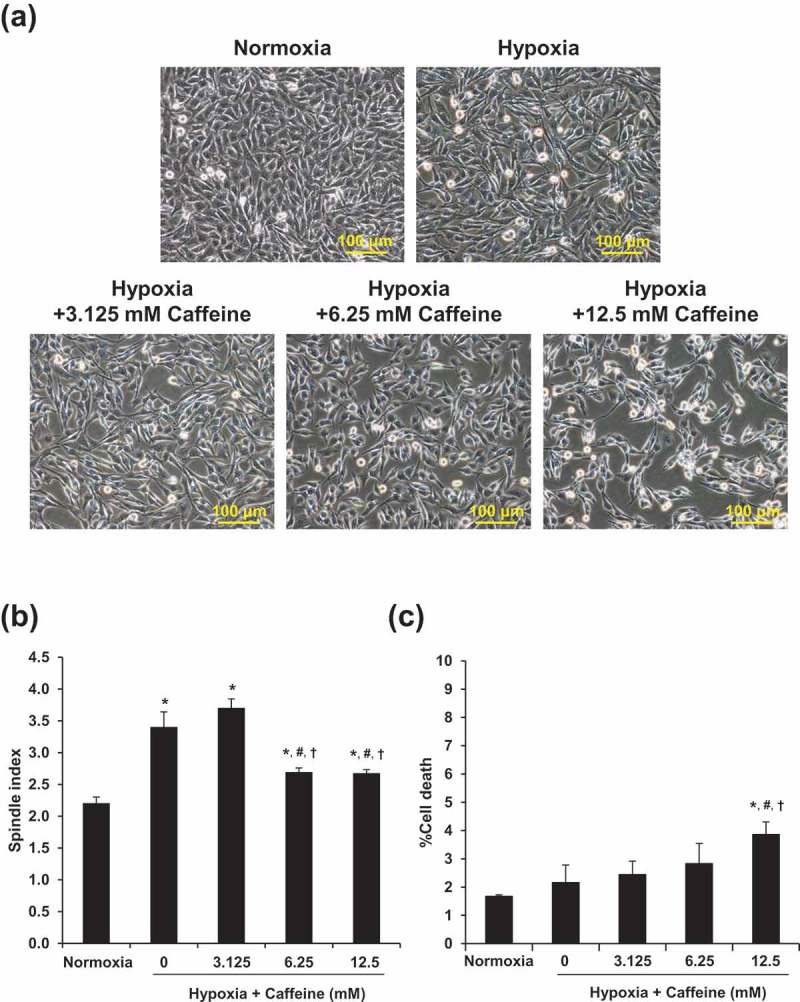
Validation of the study model. (a): Cell morphology was investigated under an inverted phase-contrast microscope (original magnification power = 200X for all panels). (b): Spindle index. (c): Percentage of cell death. Each bar represents the mean ± SEM of the data derived from three independent biological replicates. * *p* < 0.05 vs. control (normoxia); # *p* < 0.05 vs. hypoxia; †*p* < 0.05 vs. hypoxia + 3.125 mM caffeine.

To ensure that fibroblasts under hypoxic condition were activated and both hypoxia and caffeine were not too toxic to the cells (as we did not want to examine the effects of cell death), cell morphology and death were initially analyzed. After the cells were incubated under normoxia or hypoxia without or with caffeine treatment (3.125, 6.25, or 12.5 mM) for 12 h, cell morphology was examined. Comparing to the control cells under normoxia, the hypoxic cells underwent elongation (as determined by morphology and spindle index), which is one of the indicators for fibroblast activation [17,18], whereas caffeine at 6.25 and 12.5 mM significantly reduced the elongating effect of hypoxia (Figure 1(a,b)) (note that fibroblast activation was more accurately examined in subsequent experiments; see below). Cell death assay showed that hypoxia alone and hypoxia with caffeine treatment at 3.125 and 6.25 mM did not significantly affect cell death, whereas the higher dose of caffeine (at 12.5 mM) significantly increased the percentage of cell death (Figure 1(c)). Based on these data, caffeine at 6.25 mM was selected for all subsequent experiments throughout.

### Effect of hypoxia and caffeine on fibroblast activation

**Figure 2.**
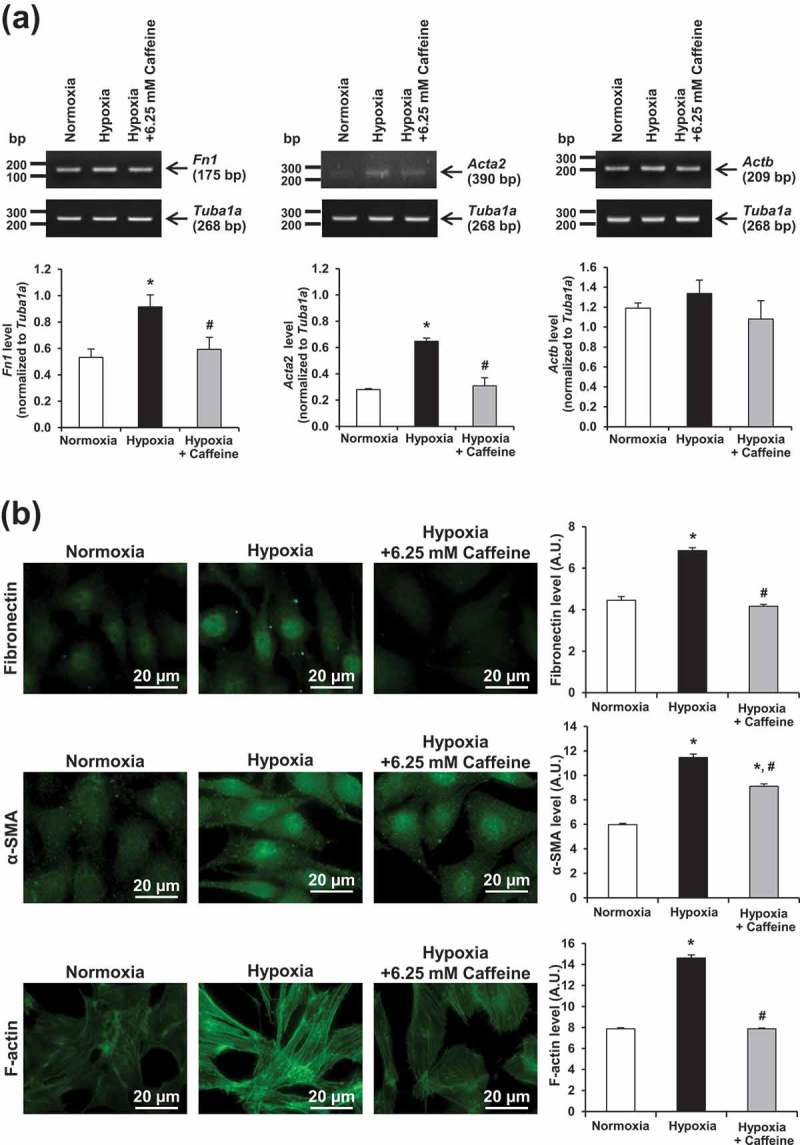
Effect of hypoxia and caffeine on fibroblast activation. Markers for fibroblast activation were evaluated at both mRNA and protein levels. (a) Semi-quantitative RT-PCR. (b) Immunofluorescence staining (original magnification power = 1000X for all panels). Fluorescence intensity was measured from at least 100 cells in ≥10 random HPF for each sample. Each bar represents the mean ± SEM of the data derived from three independent biological replicates. *p < 0.05 vs. control (normoxia); #*p* < 0.05 vs. hypoxia.

Markers for fibroblast activation, including fibronectin, α-SMA, and actin stress fiber, were examined by semi-quantitative reverse transcription-polymerase chain reaction (RT-PCR) and immunofluorescence staining. Under hypoxia, mRNA levels of fibronectin (*Fn1*) and α-SMA (*Acta2*) were significantly increased (Figure 2(a)). Caffeine at 6.25 mM could completely restore both *Fn1* and *Acta2* to their basal levels. Similarly, mRNA level of β-actin (*Actb)* tended to be increased by hypoxia and could be recovered by caffeine, but the changes did not reach the statistically significant threshold (Figure 2(a)). Immunofluorescence study demonstrated the consistent results on changes in protein levels of fibronectin and α-SMA (Figure 2(b)). In addition, actin filaments were rearranged into actin stress fibers with an increase in its total level under hypoxic condition. Caffeine showed a protective effect as evidenced by a complete recovery of both arrangement and level of actin stress filaments (Figure 2(b)).

### Effect of hypoxia and caffeine on oxidative stress

**Figure 3.**
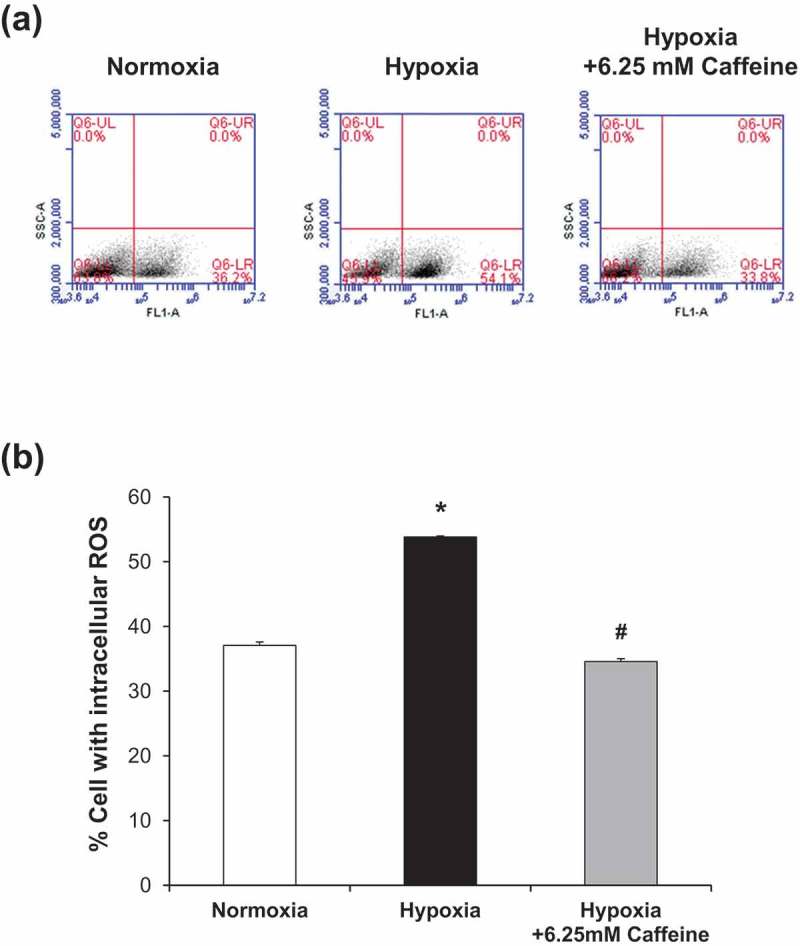
Effect of hypoxia and caffeine on intracellular ROS production. (a): Representative dot plots depicting cells with ROS analyzed by flow cytometry using DCFH-DA. (b): Quantitative analysis of ROS production in each condition. Each bar represents the mean ± SEM of the data derived from three independent biological replicates. * *p* < 0.05 vs. control (normoxia); #*p* < 0.05 vs. hypoxia.

**Figure 4.**
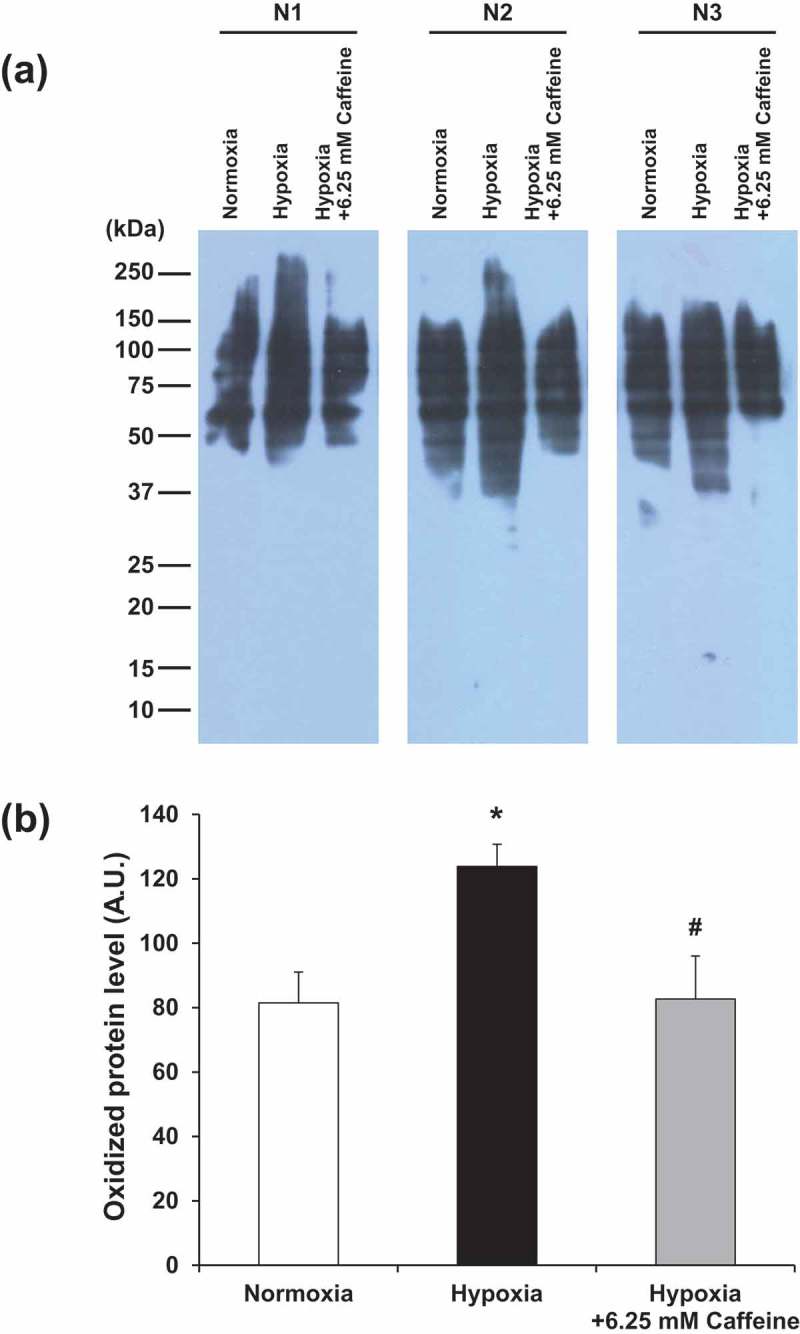
Effect of hypoxia and caffeine on levels of oxidized proteins. (a) Oxidized protein levels were determined by OxyBlot assay. (b) Quantitative analysis of band intensity of the oxidized proteins detected in each condition. Each bar represents the mean ± SEM of the data derived from three independent biological replicates. *p < 0.05 vs. control (normoxia); #*p* < 0.05 vs. hypoxia.

Markers for oxidative stress were examined by measuring intracellular reactive oxygen species (ROS) using flow cytometry and by OxyBlot assay. The results showed that intracellular ROS was significantly elevated under hypoxia, whereas 6.25 mM caffeine could completely reserve the intracellular ROS to its basal level (Figure 3). Similarly, OxyBlot analysis revealed a significant increase in levels of oxidized proteins, whereas caffeine could completely restore such modified proteins to their basal levels (Figure 4).

### Effect of hypoxia and caffeine on catalase activity

**Figure 5.**
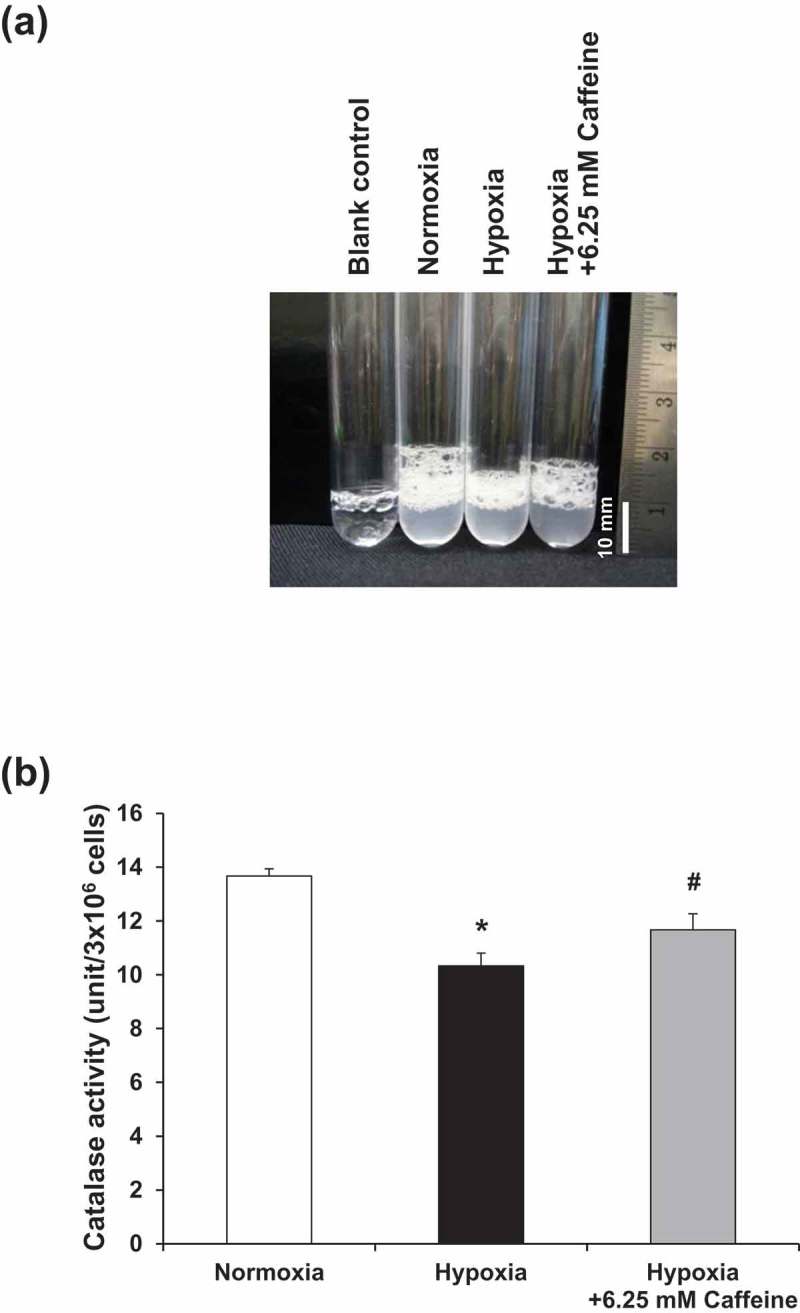
Effect of hypoxia and caffeine on catalase activity. (a) Oxygen-forming foam as a result of catalase activity to breakdown H_2_O_2_ inside individual test tubes. (b) Quantitative analysis of catalase activity in each condition. Each bar represents the mean ± SEM of the data derived from three independent biological replicates. *p < 0.05 vs. control (normoxia); #*p* < 0.05 vs. hypoxia.

The antioxidative property of caffeine was assessed by measuring catalase activity. In comparison to normoxia, catalase activity was significantly decreased (as indicated by the lower height of O_2_-forming foam) in the hypoxic cells (Figure 5). Caffeine treatment could restore the catalase activity to a level comparable to that of the basal control (Figure 5).

### Effect of hypoxia and caffeine on nuclear factor erythroid 2-related factor 2 (Nrf2) signaling

**Figure 6.**
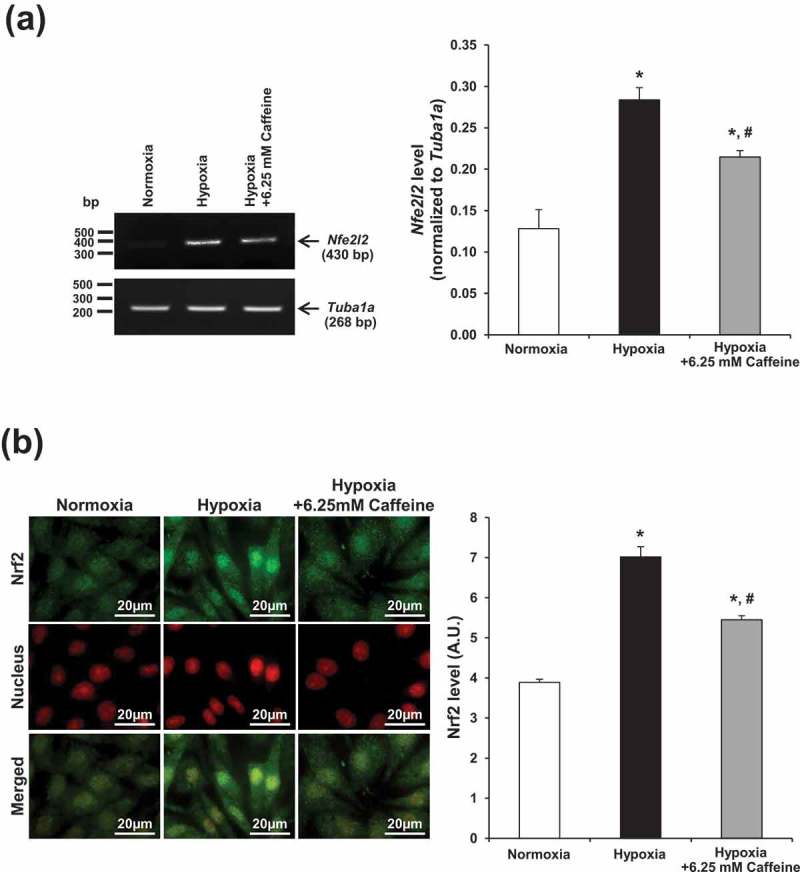
Effect of hypoxia and caffeine on Nrf2 signaling. Nrf2 was evaluated at both mRNA and protein levels. (a) Semi-quantitative RT-PCR. (b) Immunofluorescence staining of Nrf2 (green) and nuclei (red). Nuclear localization of Nrf2 is shown in the merged view (yellow). Original magnification power = 1000X for all panels. Fluorescence intensity was measured from at least 100 cells in ≥10 random HPF for each sample. Each bar represents the mean ± SEM of the data derived from three independent biological replicates. *p < 0.05 vs. control (normoxia); #*p* < 0.05 vs. hypoxia.

Nrf2 is involved in oxidative stress and plays roles as the stress-responsive signaling molecule [19]. Nrf2 mRNA (*Nfe2l2*) and protein levels were thus assessed. Semi-quantitative RT-PCR and immunofluorescence assay revealed the consistent data showing that *Nfe2l2* mRNA and Nrf2 protein were significantly increased, particularly in the nuclei, in hypoxic cells (Figure 6). These changes, however, could be partially reduced by caffeine treatment (Figure 6).

### Effect of hypoxia and caffeine on *Hif1a* gene

**Figure 7.**
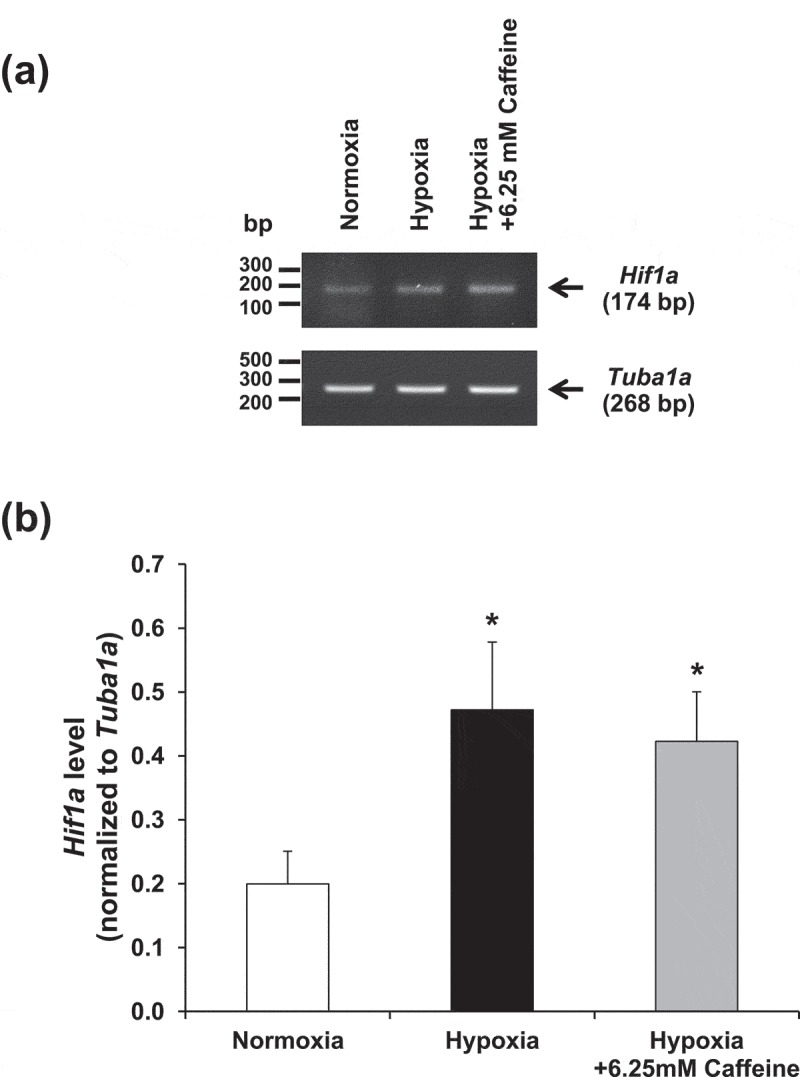
Effect of hypoxia and caffeine on *Hif1a* gene. (a) *Hif1a* mRNA expression level was examined by semi-quantitative RT-PCR. (b) Quantitative analysis of band intensity of *Hif1a* mRNA detected in each condition. Each bar represents the mean ± SEM of the data derived from three independent biological replicates. *p < 0.05 vs. control (normoxia).

*Hif1a* gene encoding hypoxia-inducible factor-1α transcription factor is a well-known target for upstream signaling of the cellular adaptive response under hypoxia [20]. We thus examined whether *Hif1a* was affected by caffeine to inhibit hypoxia-induced fibroblast activation or not. Semi-quantitative RT-PCR showed that *Hif1a* expression was upregulated by hypoxia (Figure 7). However, caffeine did not affect *Hif1a* expression, indicating that caffeine mediated its anti-fibrotic activity against hypoxia-induced renal fibroblast activation most likely through the downstream pathways (Figure 7).

## Discussion

In the present study, we have investigated the protective effect of caffeine against renal fibroblast activation induced by hypoxia. Caffeine is the well-known bioactive chemical found mainly in coffee bean that exhibits an antagonistic effect against adenosine receptors [21]. Interestingly, a number of studies have highlighted the beneficial effects of caffeine and coffee consumption on human health, including the prevention of tissue fibrosis [22,23]. A meta-analysis has revealed the lower risk of hepatic fibrosis and cirrhosis in coffee consumers [24]. In addition, caffeine itself also possesses antioxidative property that has been well documented to protect against experimental liver fibrosis [25–27]. However, the investigation of the anti-fibrotic effect of caffeine on renal fibrosis was limited.

Renal hypoxia is accepted as a key player in the progression of CKD. Almost two decades ago, the contribution of renal hypoxia to CKD had been proposed through the “chronic hypoxia hypothesis,” explaining that the loss of microvasculature creates a hypoxic environment that consequently activates the fibrotic response in tubulointerstitial cells [28]. The hypoxic insult can cause tubular epithelial cell damage, induction of inflammation, and, more importantly, fibroblast activation [29]. The characteristics of activated fibroblasts are related to augmentation of α-SMA and ECM accumulation [30]. Our findings were consistent with those reported in a previous study showing the critical role of hypoxia-induced ECM accumulation, resulting in the deposition of collagen and fibronectin [31]. In the present study, hypoxia-induced fibroblast activation was confirmed by the increment of fibroblast activation markers, including fibronectin and α-SMA. However, these features were abolished by caffeine treatment. These observations were supported by the previous studies that revealed the inhibitory effect of caffeine on the fibrotic process as indicated by the decreased α-SMA and collagen content and the restored glutathione peroxidase level in the damaged liver [32,33].

In addition, it is known that hypoxia could affect actin dynamics and thus leads to alterations in cell morphology, movement, and adhesion [34,35]. Under hypoxia, actin dynamics, including the rearrangement of actin filaments into stress fibers, is mediated by Rho GTPase signaling pathway [36]. In our present study, actin stress fiber assembly was significantly increased under hypoxia that could be abolished by caffeine treatment, implicating that such inhibitory or attenuation effect on actin stress fibers might be mediated through Rho GTPase pathway. Collectively, these findings suggest that caffeine could alleviate characteristics of the activated fibroblast induced by hypoxia.

Besides the induction of activated fibroblast markers, mitochondrial adaptation in response to hypoxic condition can cause reductive carboxylation, which leads to ROS overproduction and subsequently oxidative stress [37]. The dangerous oxidative radicals (or ROS) could be balanced by antioxidants derived from many natural compounds [38]. Caffeine and its metabolites, 1-methylxanthine and 1-methyluric acid, are considered the natural chemicals with the potent antioxidant properties [39]. It has been reported that caffeine could modulate ROS generation in a model of pulmonary oxygen toxicity [40]. In addition, caffeine could protect human skin fibroblasts against H_2_O_2_-induced necrosis [41]. Moreover, both in *vitro* and in *vivo* models have revealed that caffeine could inhibit ROS overproduction, resulting in reduction of atherosclerosis [42]. In concordance, we found that caffeine treatment significantly abolished hypoxia-induced ROS overproduction in activated fibroblasts. Since ROS could promote protein carbonylation (also known as protein oxidation) by direct oxidation of amino acid side chain (i.e. Lys, Arg, Pro, Thr, and Cys), leading to the formation of reactive aldehyde and ketone [43], we thus investigated levels of oxidized proteins induced by hypoxia. The more ROS generated under hypoxia, the greater levels of oxidized proteins observed. However, caffeine treatment could diminish such effect. These results supported that caffeine has the protective effect against hypoxia-induced ROS overproduction.

During oxidative stress, defense systems are generally activated to eliminate or improve the damages caused by intracellular ROS [44]. Theoretically, antioxidants can react directly with radicals via donating or accepting an electron to maintain the redox balance. In addition, the antioxidants can also indirectly remove stressful radicals by enhancing the expression and activity of many antioxidant enzymes [45,46]. Catalase is one of such antioxidant enzymes required for ROS homeostasis that can protect cellular redox imbalance by converting hydrogen peroxide (H_2_O_2_) into water (H_2_O) and oxygen (O_2_) [47,48]. Our data showed that catalase activity was significantly dropped under hypoxic condition. However, the antioxidant property of caffeine, to some extent, could restore catalase activity that was suppressed by hypoxia. These findings were consistent with the liver fibrosis model demonstrating that hepatoprotective effects of caffeine against liver fibrotic changes involved many mechanisms, such as inhibition of Snail1, downregulation of pro-fibrotic genes, and activation of antioxidant enzymes including catalase [26]. However, it should be noted that the decline of catalase could not be completely recovered by caffeine. Therefore, there should be other antioxidant mechanisms that also play roles for such renoprotective effects of caffeine that deserve further investigations.

Nrf2 is a major transcriptional factor that plays a vital role in stress-responsive pathway [19]. Nrf2 is normally controlled via negative regulator, Kelch-like ECH-associated protein 1 (Keap1)-culllin3 (Cul3) system, which marks Nrf2 for proteasomal degradation. During oxidative stress, Nrf2 is activated via conformational change of Keap1, leading to detachment of Keap1–Nrf2 complex, which subsequently allows nuclear translocation of Nrf2 [49]. Previous evidence has shown the synergistic effect of the increased level of Nrf2 and hypoxia-induced factor-2α to switch on transcription of cytoprotective genes in order to withstand hypoxic condition [50]. In concordance, our data revealed that hypoxia upregulated Nrf2 level, particularly in the nuclei, whereas caffeine could partially restore Nrf2 level. This result might attribute to Nrf2 activation induced by ROS, which could modify redox status of Keap1 through cysteine residue to become oxidized form that allowed Nrf2 activation afterward, thereby cell survival during hypoxic condition [51,52]. In consistent with the effect on catalase activity, change in Nrf2 expression was not completely recovered by caffeine. Again, there should be other antioxidant mechanisms than catalse/Nrf2 pathways that also play roles in renoprotection by caffeine during hypoxia. It should be noted that while catalase was decreased, Nrf2 was upregulated by hypoxia. It was thus possible that the sustained hypoxia in our experimental condition caused exhaustion of various antioxidant pathways, leading to accumulation of the intracellular ROS. Nrf2 was then activated to compensate or cope with such oxidative stress. When caffeine recovered the destructive effects of hypoxia, the upregulated Nrf2 then gradually declined.

In summary, this study revealed that hypoxia induced renal fibroblast activation that could be attenuated by caffeine. The protective effect of caffeine against fibroblast activation was likely due to its antioxidant property to reduce intracellular ROS generated in response to hypoxic condition. While caffeine did not affect *Hif1a* gene expression, the cellular mechanisms underlying such protective and antioxidative effects of caffeine most likely mediated, at least in part, through downstream catalase and Nrf2 pathways.

## Materials and methods

### Cell culture

Baby hamster kidney (BHK-21) fibroblast cell line (ATCC, Manassas, VA) was used as an *in vitro* model to examine the effects of hypoxia and caffeine on renal fibrogenesis. The renal fibroblast cells were grown and maintained in a complete medium containing Eagle’s minimum essential medium (Gibco, Grand Island, NY), supplemented with 10% heat-inactivated fetal bovine serum (Gibco), 60 U/ml penicillin G, and 60 µg/ml streptomycin (Sigma–Aldrich, St. Louis, MO) in a humidified incubator with 5% CO_2_ at 37°C.

### Hypoxia induction and caffeine treatment

For hypoxic experiments, the cell monolayers in culture dishes (6 × 10^5^ cells/dish) were transferred into a hypoxic chamber (Stemcell Technologies Inc., Vancouver, Canada) containing premixed gas (1% O_2_, 5% CO_2_, and 94% N_2_) for 12 h. The temperature inside the chamber was set at 37°C, and the chamber was moistened with a dish containing sterile water. In parallel, the cells cultured in a humidified incubator with 21% O_2_ and 5% CO_2_ at 37°C served as the control (normoxia). To evaluate the effect of caffeine during hypoxia, the cells cultured under hypoxic condition were co-treated with various doses (3.125, 6.25, and 12.5 mM) of caffeine (Sigma–Aldrich).

### Cell morphology and spindle index

Effects of hypoxia and caffeine on cell morphology were examined under a phase-contrast microscope (Olympus CKX41) (Olympus Co. Ltd., Tokyo, Japan). Spindle index was calculated from at least 100 cells in ≥10 random high-power fields (HPF) of each sample by using the following formula:

*Formula 1*:

Spindle index=length of each cell/width of each cell

### Cell death assay

Trypan blue exclusion assay was used to examine cell death. The cell monolayers were harvested by trypsinization using 0.1% trypsin in 2.5 mM ethylenediaminetetraacetic acid (EDTA)/phosphate buffered saline (PBS) in combination with all floating cells, followed by centrifugation at 1,000x *g* for 5 min. Thereafter, the cells were immediately resuspended in PBS and stained with 0.4% trypan blue solution (Gibco). Total and dead cells were counted using a hemacytometer. The percentage of cell death was calculated as follows:

*Formula 2*:

$$\%\;Cell\;death=\left(Number\;of\;dead\;cells/Total\;\;cell\;number\right)\;x\;100\%$$

### Semi-quantitative RT-PCR

Total RNA was extracted using Trizol reagent (Invitrogen, Eugene, OR) and Direct-zol RNA MiniPrep (Zymo Research, Irvine, CA). An equal amount of total RNA was used for the preparation of cDNA using SuperScript III reverse transcriptase (Invitrogen). Semi-quantitative PCR was performed using Taq DNA polymerase (New England BioLabs, Beverly, MA) to assess expression levels of *Fn1, Acta2, Actb, Nfe2l2, Hif1a*, and *Tuba1a*. Amplifications were carried out using the following primers: *Fn1* forward: 5ʹ-CTGAAGAGACTTGCTTTGAC-3ʹ, reverse: 5ʹ-CCAATCTTGTAGGACTGACC-3ʹ; *Acta2* forward: 5ʹ-ACTACTGCTGAGCGTGAGAT-3ʹ, reverse: 5ʹ-TCACACTTCATGATGCTG-3ʹ; *Actb* forward: 5ʹ-TTTGAGACCTTCAACACCC-3ʹ, reverse: 5ʹ-AGGATCTTCATGAGGTAGTC-3ʹ; *Nfe2l2* forward: 5ʹ-GCCTTTTTCGCTCAGTTAC-3ʹ, reverse: 5ʹTGGTAGTCTCAACCAGC-3ʹ; *Hif1a* forward: 5ʹ-AATCGGCGACCCCAGTG-3ʹ, reverse: 5ʹ-GAGAGGAAGAGGGAGCCTCG-3ʹ; and *Tuba1a* forward: 5ʹ-CGCCCAACCTACACTAATCTAA-3ʹ, reverse 5ʹ-CATGGCGAGGGTCACATTTC-3ʹ. The PCR products of *Fn1, Acta2, Actb, Nfe2l2, Hif1a*, and *Tuba1a* were 175, 390, 209, 430, 174, and 268 bp, respectively. The PCR reaction was started with an initial DNA denaturation step (at 95°C for 4 min) followed by 25 cycles (for *Fn1, Actb*, and *Nfe2l2*) or 30 cycles (for *Acta2, Hif1a*, and *Tuba1a*) of denaturation at 95°C for 30 s, annealing at 56°C for 30 s, extension at 68°C for 30 s, and a final extension step at 68°C for 5 min. PCR products were then resolved by 1% agarose gel electrophoresis and stained by RedSafe (iNtRON Biotechnology, Inc., Seongnam, South Korea). The DNA bands were visualized using ChemiDoc MP Imaging System (Bio-Rad, Berkeley, CA) and quantitated by using ImageQuant TL software (GE Healthcare, Uppsala, Sweden).

### Immunofluorescence staining

Renal fibroblasts were grown on coverslips and treated as described above. Immunofluorescence staining was performed as described previously [53,54]. Briefly, the cells were washed with membrane-preserving buffer (1 mM MgCl_2_ and 0.1 mM CaCl_2_ in PBS), fixed with 4% paraformaldehyde/PBS for 15 min, and permeabilized with 0.1% triton-X100/PBS for 15 min at 25°C. After extensive washing with membrane-preserving buffer, non-specific bindings were blocked with 5% bovine serum albumin (BSA)/PBS at 25°C for 30 min, and the cells were then probed with mouse monoclonal anti-fibronectin (Santa Cruz Biotechnology, Santa Cruz, CA), mouse monoclonal anti-α-SMA (Santa Cruz Biotechnology), or rabbit polyclonal anti-Nrf2 antibody (Santa Cruz Biotechnology) (all were diluted 1:50 in 1% BSA/PBS) at 4°C overnight. After washing, the cells were further incubated with corresponding fluorescence-conjugated secondary antibody (Dako, Glostrup, Denmark) (diluted 1:500 in 1% BSA/PBS) at 25°C for 1 h. To examine actin stress fiber formation, the cells were incubated with Oregon green-conjugated-phalloidin (diluted 1:50 in 1% BSA/PBS) (Invitrogen – Molecular Probes, Burlington, Canada) at 25°C for 1 h. The nuclei were counterstained with Hoechst dye (Invitrogen – Molecular Probes) (diluted 1:1,000 in 1% BSA/PBS) or 0.15 µg/ml propidium iodide (BD Biosciences, San Jose, CA). Finally, the coverslips were then mounted onto glass slides using 50% glycerol/PBS, and the fluorescence images were captured under Nikon Eclipse 80i fluorescence microscope (Nikon, Tokyo, Japan). Quantitative data were obtained and analyzed from at least 100 cells in ≥10 random HPFs for each sample using NIS-Elements D V.4.11 (Nikon).

### Analysis of intracellular ROS by flow cytometry

Intracellular ROS production was determined by using 2ʹ,7ʹ-dichlorodihydrofluorescein diacetate (DCFH-DA) and analyzed by flow cytometry. Briefly, the cells were collected by trypsinization as suspension and incubated with 5 μM DCFH-DA (Invitrogen) for 30 min on ice prior to analysis by BD Accuri C6 flow cytometer (BD Accuri) (Beckman Coulter, Fullerton, CA).

### Measurement of levels of oxidized proteins by OxyBlot assay

Oxidized proteins were detected by OxyBlot assay using the OxyBlot protein oxidation detection kit (Merck Millipore, Darmstadt, Germany) as previously described [55,56]. Briefly, proteins (with an equal amount) derived from each sample were derivatized with 2,4-dinitrophenylhydrazine (DNPH), resolved by 12% SDS-PAGE, and transferred onto a nitrocellulose membrane. Non-specific bindings were blocked with 5% skim milk/PBS for 1 h at 25°C. The membrane was then incubated with a rabbit polyclonal anti-dinitrophenyl (DNP) antibody (diluted 1:500 in 1% BSA/PBS) at 4°C overnight, followed by a corresponding secondary antibody conjugated with horseradish peroxidase (Dako) (diluted 1:1,000 in 1% BSA/PBS) at 25°C for 1 h. After washing with PBS three times, the immunoreactive protein bands were developed by SuperSignal West Pico chemiluminescence substrate (Pierce Biotechnology, Rockford, IL) and visualized by autoradiogram.

### Measurement of catalase activity

Catalase activity was measured to determine the antioxidant activity of the cells according to the protocol described previously [57]. Briefly, the cells were harvested by trypsinization and adjusted to 1 × 10^7^ cells/condition in 100 µl PBS in a test tube. The cell suspension was then mixed with 100 µl of 1% triton X-100 and 100 µl of 30% (w/v) hydrogen peroxide (H_2_O_2_) (Fischer Scientific, Leicestershire, UK) and incubated at 25°C for 15 min. The height of O_2_-forming foam produced by the breakdown of H_2_O_2_ by catalase was measured to indicate the enzymatic activity of catalase.

### Statistical analysis

All quantitative data were derived from at least three independent biological replicates and are presented as mean ± SEM. Multiple comparisons were done by using one-way ANOVA with Tukey’s honestly significant difference (HSD) post-hoc test. *p* Values <0.05 were considered statistically significant.
